# Automatic Disease Detection from Strawberry Leaf Based on Improved YOLOv8

**DOI:** 10.3390/plants13182556

**Published:** 2024-09-11

**Authors:** Yuelong He, Yunfeng Peng, Chuyong Wei, Yuda Zheng, Changcai Yang, Tengyue Zou

**Affiliations:** 1 College of Mechanical and Electrical Engineering, Fujian Agriculture and Forestry University, Fuzhou 350002, Chinachuyongwei2021@163.com (C.W.);; 2 Fujian Key Laboratory of Agricultural Information Sensoring Technology, Fujian Agriculture and Forestry University, Fuzhou 350002, China; 3 College of Computer and Information Sciences, Fujian Agriculture and Forestry University, Fuzhou 350002, China

**Keywords:** deep learning, smart agriculture, strawberry disease, target detection

## Abstract

Strawberries are susceptible to various diseases during their growth, and leaves may show signs of diseases as a response. Given that these diseases generate yield loss and compromise the quality of strawberries, timely detection is imperative. To automatically identify diseases in strawberry leaves, a KTD-YOLOv8 model is introduced to enhance both accuracy and speed. The KernelWarehouse convolution is employed to replace the traditional component in the backbone of the YOLOv8 to reduce the computational complexity. In addition, the Triplet Attention mechanism is added to fully extract and fuse multi-scale features. Furthermore, a parameter-sharing diverse branch block (DBB) sharing head is constructed to improve the model’s target processing ability at different spatial scales and increase its accuracy without adding too much calculation. The experimental results show that, compared with the original YOLOv8, the proposed KTD-YOLOv8 increases the average accuracy by 2.8% and reduces the floating-point calculation by 38.5%. It provides a new option to guide the intelligent plant monitoring system and precision pesticide spraying system during the growth of strawberry plants.

## 1. Introduction

The commercial strawberry production has a high economic value [1]. However, strawberry plants are susceptible to various diseases, among which leaf diseases [2] are particularly common. These diseases seriously reduce the yield and quality of strawberry production and may cause substantial economic losses [3]. Because the wide variety of diseases and complex plant pathogenesis make it hard to prevent and control the diseases, traditionally, growers’ visual inspections are relied upon to detect plant diseases in the early stage [4]. However, because strawberries are affected by many types of diseases that share similarities [5], visual inspection may result in misdetection or misjudgment [6]. With the advancement of the image-processing technology and an increase in computational power [7], more and more researchers are paying attention to techniques for the intelligent diagnosis of crop diseases based on image-processing technology [8]. These techniques not only save time and reduce labor costs but also are more precise and accurate [9]. Consequently, the employment of image recognition technology to achieve an efficient and timely detection of strawberry diseases [10] will boost strawberry production, lead to a more precise application of pesticides, and collect more reliable data for the agricultural administration [11,12,13].

In recent years, machine learning has gradually become a common approach in the plant disease detection field [14]. K-means clustering was used to separate the disease-affected area of the grape leaf from the healthy area, and features were extracted based on three color models: RGB, HSV, and l*a*b. Then, the support vector machine (SVM) was used to diagnose and classify the leaf diseases, achieving an accuracy of 98.71% [15]. K-Nearest Neighbor (KNN) classifier was also adopted to recognize the affected maize plant leaves with an accuracy of 90% [16]. However, these methods rely on agricultural experts’ experience to extract disease features, which is time-consuming and has limited recognition accuracy. Compared with traditional methods, deep learning techniques can shorten the recognition time and improve the recognition accuracy [17]. Furthermore, DenseNet201 and SVM can be used together to recognize diseases on maize leaves and achieve a recognition rate of 94.9% [18]. To detect small-target lepidoptera pests on leaves, MD-YOLO was proposed. The DenseNet block and Adaptive attention module were added to the feature extraction process, and the accuracy could reach 86.2% [19]. The Updated Faster R-CNN architecture was proposed [20] for the automatic detection of the beet leaf spot disease and achieved an overall correct classification rate of 95.48%, with 155 images being processed.

For the detection of strawberry diseases, a new Faster R-CNN architecture was proposed to deal with the complex background and small lesions in images of strawberry diseases [21]. Its model can effectively extract abundant image features of strawberry diseases and reach an average accuracy of 92.18% on their self-built dataset. Four convolutional neural network models (SqueezeNet, EfficientNet-B3, VGG-16, and AlexNet) were trained to classify healthy and leaf scorch-infected strawberry plants [22], and the EfficientNet-B3 model achieved the highest accuracy of 80% among these four models. Moreover, the DAC-YOLOv4 was proposed to detect powdery mildew and infected leaves of strawberries under complex backgrounds [23]. The original YOLOv4 backbone and neck are replaced with deep convolution and a hybrid attention mechanism. By using a combination of convolutional block layer (CBL) and depthwise-convolutional block layer (DW-CBL) structures to simplify the convolution, the DAC-YOLOv4 can achieve an average accuracy of 72.7%.

These studies provide good ideas for image-based crop disease detection. However, there are still some actual problems in strawberry disease detection under real-world conditions: (1) One problem is the poor quality of training datasets. Most existing training datasets in this field are derived from single background images in the laboratory. It is difficult for the model trained with these kinds of training sets to adapt to recognition tasks under real-world complex conditions. (2) Another problem is insufficient accuracy. The differences between the features of some specific strawberry leaf diseases are indistinguishable. They show a high degree of similarity, which makes it difficult for the neural network to classify them, resulting in a lower recognition accuracy. (3) Huge computational complexity is a challenge. The leaf disease area is small, and its symptoms are not obvious. Thus, a large feature space for classification is needed. This requires a large number of computing resources and is hard to be deployed on a mobile device. 

To solve these problems, the following key improvements are added to the original YOLOv8 for strawberry leaf disease detection: (1) KernelWarehouse [24] dynamic convolution is taken to replace the original convolutions in the backbone network and in the C2f (CSPLayer_2ConV) function. Because KWConv replaces Conv in the C2f function, the replaced module is named KW-C2f. This module can extract the texture information of leaf diseases more efficiently and reduce the computational complexity significantly. (2) The Triplet Attention mechanism is adopted [25], which enhances the feature extraction ability of the model at different spatial scales and further significantly improves the accuracy. (3) A parameter-sharing DBB (diverse branch block [26]) sharing head is constructed to help the model extract the feature information more efficiently without significantly increasing the computational complexity. In the dataset, we collected pictures of strawberry diseases in real-life plantations and increased the generalization of the dataset via data enhancement. Finally, ablation studies were carried out, and experiments were conducted to confirm the performance of the algorithm. The results show that the introduced algorithm performs better in strawberry disease detection in complex real-world circumstances. After engineering optimization, the proposed algorithm can be deployed on a self-propelled robot in the field with a digital camera. Using the real-time video taken from the camera, the strawberry disease can be discovered.

## 2. Results and Discussion

The most common evaluation metrics in deep learning were selected to evaluate the algorithm: precision (P), recall (R), mean average precision (mAP), and floating-point operations (FLOPS). The rate of precision is the proportion of true victimizations out of all results detected as victimizations. Recall is the proportion of detected victimizations to the total number of actual victimizations. The average precision (AP) is calculated based on precision and recall. The area under the precision–recall (P–R) curve is the AP value. mAP is the average value of all the categories of precision. It is the main evaluation index for target detection, which reflects the model’s overall performance. The higher the value, the better the model performs. FLOPS are used to measure the running time of the model. The lower the value, the less calculation the model needs to perform. Furthermore, mAP@0.5 indicates the mAP value when the Intersection over Union (IoU) is set to 0.5. The formulas are shown in Equations (1) and (2).

(1) $$\mathrm{AP}=\int_{0}^{1}P(R)\mathrm{dR}$$

(2) $$\mathrm{mAP}=\frac{1}{n}\sum_{i=1}^{n}\mathrm{AP}$$

**Figure 1:**
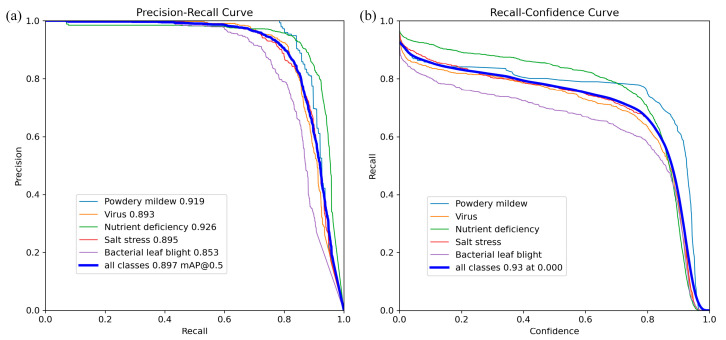
Performance curves of the KTD-YOLOV8 results. (**a**) Precision–recall Curve; (**b**) Recall–confidence Curve.

**Figure 2:**
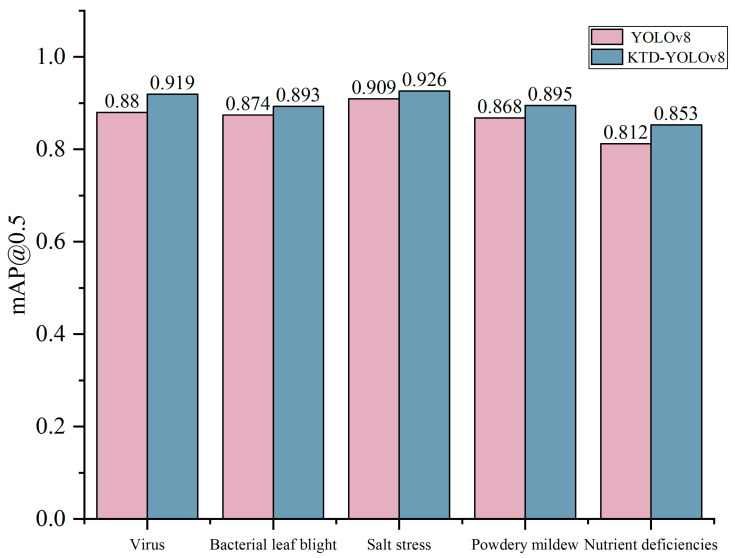
A comparison of the accuracy of YOLOv8 and KTD-YOLOv8 for various diseases.

In the overall recognition experiments, the testing dataset was inputted into the introduced KTD-YOLOv8 model, and the results are shown in Figure 1 and Figure 2. The precision of the method was 0.90, and the recall was 0.813. Its mAP@0.5 was 0.897, and the calculation amount was 17.7 GFLOPS. The results show that the KTD-YOLOv8 increases the confidence level, which can effectively increase detection accuracy in the actual environment and reduce the computation amount. 

**Figure 3:**
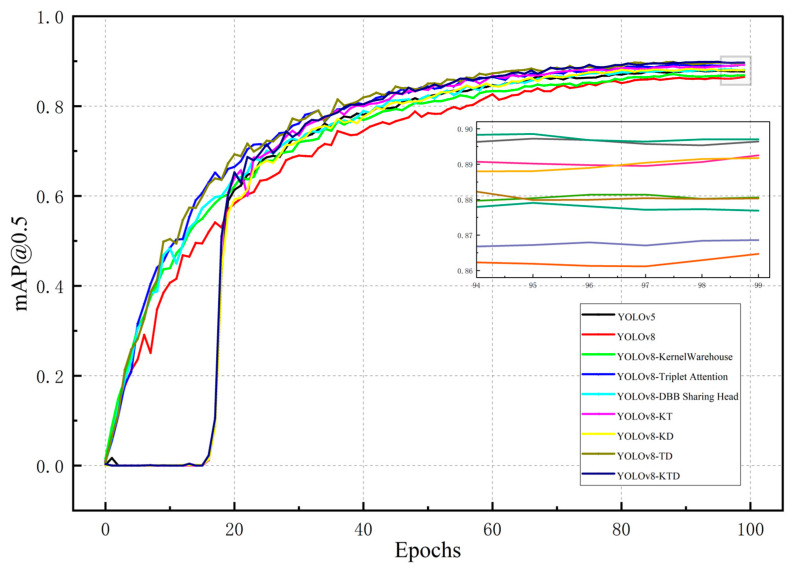
Comparison of mAP@0.5 of models.

**Figure 4:**
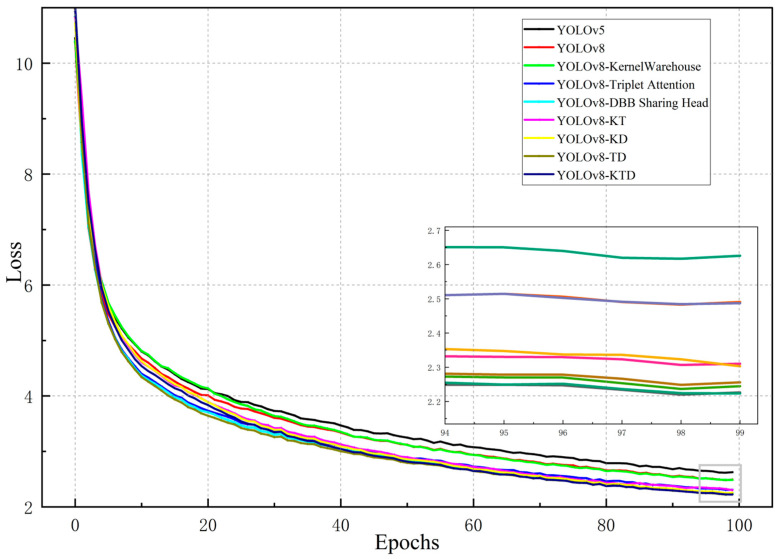
Overall loss comparison of models.

To further verify the effectiveness of individual modules in KTD-YOLOv8, the YOLOv8s model was used as the baseline model as a comparison. The ablation experiments were executed on the dataset by adding modules to the baseline network one by one, and the results are shown in Table 1 and Figure 3 and Figure 4. Compared with the original YOLOv8s model, the addition of the Triplet Attention mechanism makes the most significant contribution to the improvement of the accuracy, as mAP@0.5 increased by 2.4%, while a reduction in the computational load of 0.3 GFLOPS was achieved. The constructed DBB sharing head makes the most significant contribution to the model inference time, reducing it from 13.1 ms to 11.1 ms. It also increases the mAP@0.5 value by 1.2%, with an increase in the computational load and the number of parameters. Using KernelWarehouse to replace the base convolution reduces the computational load by 14.6 GFLOPS, and mAP@0.5 also increases by 1%. Figure 3 shows the mAP@0.5 curves for that experiment. The overall loss comparison is shown in Figure 4.

In real strawberry leaves, some spots of strawberry diseases occupy a tiny portion of the leaf. Thus, a baseline 3 × 3 convolution kernel cannot extract enough information from the target, while larger convolution kernels may cost more computational resources and ignore the tiny details. The KWConv convolution mechanism is introduced to improve it by linearly mixing several static convolutional kernels together. It increases the number of kernels and reduces the dimension for each kernel to significantly cut down the amount of computation and improve accuracy. To evaluate the effectiveness of the mechanism, the experiments were executed involving DySnakeConv [27], SPDConv [28], and KWConv. The baseline convolution component was replaced with these algorithms one by one, and calculations were run on a self-constructed dataset. The training environmental parameters were the same as before. The experimental results are shown in Table 2. It could be found that replacing the base convolution with SPDConv acquires the highest mAP@0.5 value, which reaches 88.1%. However, it increases the calculations significantly to 43 GFLOPS. Replacing the base convolution with the KWConv increases the mAP@0.5 to 87.9%, and the calculation requirement is only 14.2 GFLOPS.

To verify the effectiveness of the constructed DBB sharing head, Pose Head [29] and Aux head [30] modules were selected for comparison. In the actual environment, disease areas are often confused with the background. Thus, the detection head needs to output a large amount of predicting information. The original YOLOv8 has a decoupled head structure, so it occupies a large portion of the calculation for the whole model. To obtain higher accuracy without significantly increasing the computational complexity, the DBB sharing head was constructed in this study. The experimental results are shown in Table 3. It can be found that the Aux Head, the Pose Head, and the DBB sharing head have a nearly equal effect on improving the model, with about an 88% mAP@0.5 value. According to their calculation needs, the DBB sharing head has the advantage of saving system resources. 

The attention mechanism can help the model distinguish similar diseases. The Triplet Attention mechanism introduced in this study can integrate information in different dimensions through a series of rotations and arrangements to better capture the intrinsic characteristics of the disease area. Other mechanisms, such as SimAM [31] and CPCA [32], were added to the experiment for comparison. The attention modules were inserted into the module one by one in the experiment. The experimental parameters are the same as before. According to the experimental results shown in Table 4, adding the SimAM reduces the calculations of the model to 28.4 GFLOPS, but the mAP@0.5 was not significantly improved. Triplet Attention increases the mAP@0.5 to 89.3%, and the calculations of the model were reduced to 28.5 GFLOPS, which was only 0.1 GFLOPS more than adding the SimAM. The experiment proved that the Triplet Attention mechanism has good actual performance in strawberry disease detection. 

**Figure 5:**
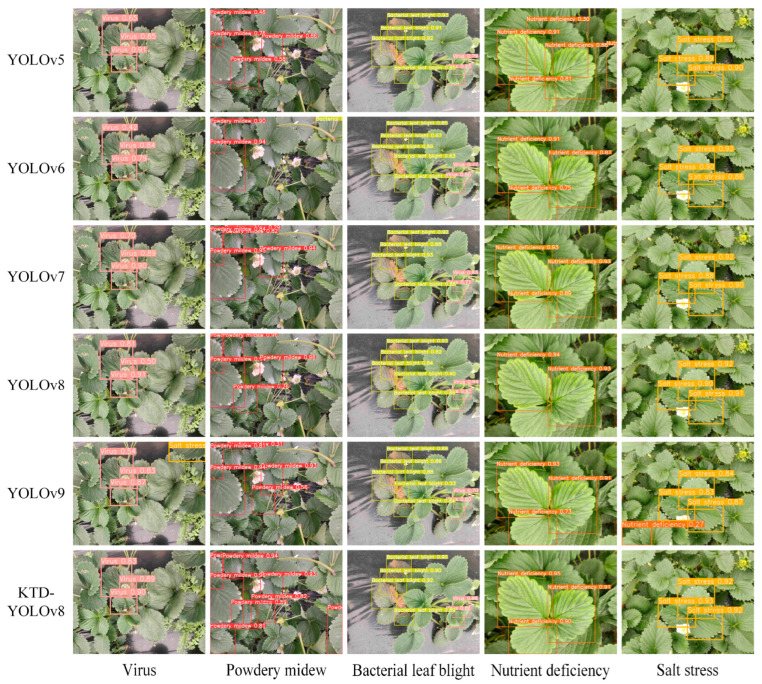
A comparison of detection effects of different detection models.

In order to further validate the detection effect of KTD-YOLOv8, the algorithm was compared with YOLOv5, YOLOv6, YOLOv7, YOLOv8, and YOLOv9 in experiments, and the results are shown in Table 5. Under the 100 training rounds, the results show that YOLOv5, YOLOv8, and KTD-YOLOv8 did not increase the parameters much. Among the three algorithms above, KTD-YOLOv8 could acquire the highest mAP@0.5 value of 89.7% and the minimum inference time of 12.1 ms. Thus, KTD-YOLOv8 showed the best comprehensive performance. Furthermore, these five algorithms and KTD-YOLOv8 were used to detect five kinds of strawberry diseases, and the comparative results are shown in Figure 5. It could be found that the KTD-YOLOv8 was able to detect the target more accurately and had a higher average confidence rate than other algorithms. In the detection test of powdery mildew with small disease characteristics and overlapping leaves, there were many missed objects in the response of YOLOv5, YOLOv6, YOLOv7, and YOLOv8 models and one missed object in YOLOv9, while KTD-YOLOv8 did not miss any detections. In the detection of bacterial leaf blight under a complex background, YOLOv9 showed false detection of wilted leaves, while KTD-YOLOv8 showed good robustness. KTD-YOLOv8 also did better in recognizing the infested leaves from the disease scene and obtained a high confidence level. 

Through experiments, it was found that the improved KTD-YOLOv8 increases the model’s mAP@0.5 by 2.8% and reduces the model’s computational load by 11.1 GFLOPS, as shown in Table 1. The introduced KW-Conv component was found to reduce the model’s computational load by 14.6 GFLOPS and increase mAP@0.5 by 1%. This was mainly due to the reduction of the kernel dimension and the increase in kernel numbers, as well as the enhancement of the dependence of convolution parameters within the same layer and between consecutive layers. As shown in Table 4, the Triplet Attention mechanism increases the mAP@0.5 by 2.4% and reduces the model’s computational load by 0.3 GFLOPS. It integrates multi-scale features better and improves extraction ability for small targets. As shown in Table 1, the DBB sharing head improves the mAP@0.5 value of the baseline model by 1.2% and decreases the model’s inference time. However, it increases the computational load by 3 GFLOPS and the number of parameters by 0.214 × 10^7^. This is because the DBB module adopts a multi-branch structure during training and uses the fusion of multiple branches as the main one during the inference procedure.

## 3. Materials and Methods

### 3.1. Image Dataset

**Figure 6:**
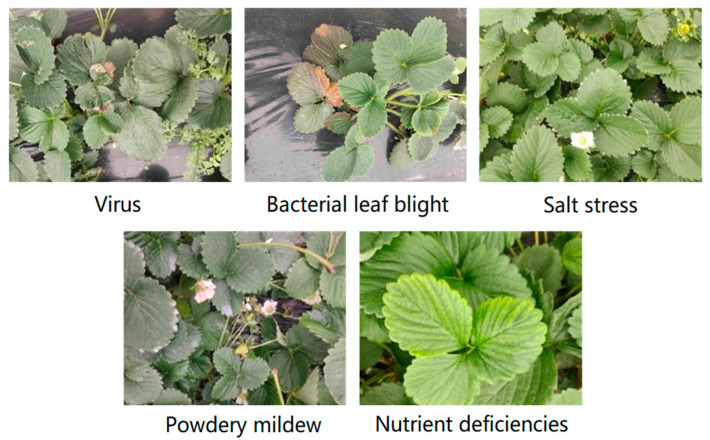
The samples of different types of diseases.

The dataset in this study was collected from a strawberry orchard in Ganzhou City, Jiangxi Province, China, between 2:00 and 5:00 p.m. on 9–24 February 2023. The time was during the dormant period of strawberries, and there were many strawberry leaves. The strawberry leaves were photographed at a distance of 40 cm. After the screening, 823 images indicating strawberry leaf disease information comprised the dataset. The dataset contained five kinds of leaf diseases. The samples are shown in Figure 6.

### 3.2. Image Enhancement

**Figure 7:**
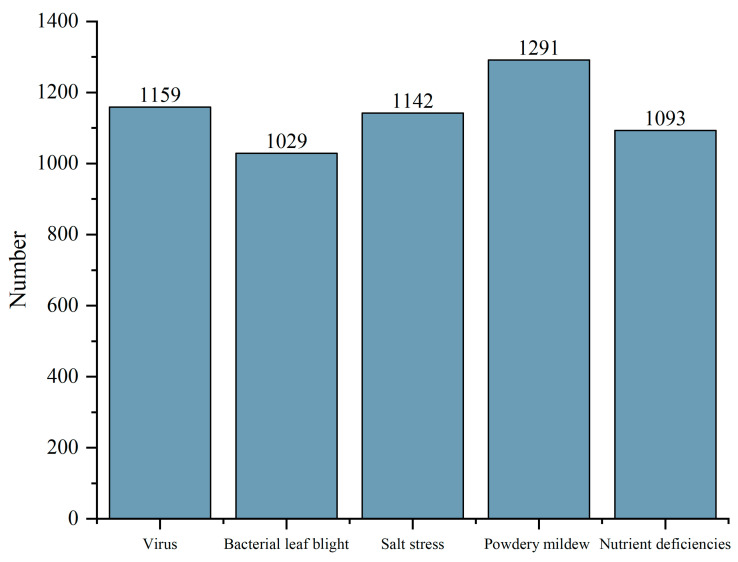
The number of images for different types of diseases.

Few training images may lead to overfitting or non-convergence of the deep learning algorithm. Therefore, we increased the number of images in the dataset to overcome this defect through image enhancement. We labeled the dataset with LabelImg software (version: 1.8.6) and generated “XML” files according to the PASCAL VOC [33] dataset format. Then, we processed the images with image-augmentation software using two randomly selected methods from the general functions for image enhancement, including rotating the images from multiple angles, adjusting the brightness, blurring, adding Gaussian noise, etc. A total of 5714 images were obtained through this enhancement, and the dataset was divided according to the ratio of 6:2:2, where 3428 images were put in the training subset, 1143 images in the validation subset, and 1143 images in the testing subset. The number of images for different types of diseases is shown in Figure 7.

### 3.3. Experimental Platform

The experimental platform used the SGD optimizer, and the training round was set to 100 epochs. The input image size was 640 × 640 pixels. The learning rate was set to 0.01, and the batch size was 16. To avoid overfitting and improve the model’s generalization ability, the mosaic data augmentation was set to the default optimal value of 1 for YOLOv8, and the mix-up was not used. To ensure the reliability and accuracy of the experimental results, all experiments were trained without using pre-trained weights. The operating system was Windows 10, using Intel(R) Core(TM) i5-12400F CPU, RTX3060 GPU, and the Pytorch 2.1.1 and CUDA 12.3 deep learning frameworks. The pre-training weights provided officially by YOLOv8s were utilized as the training initialization parameter., Under this experimental platform, the training time of the original YOLOv8s under this experimental platform was 146 min, and the number of parameters was 1.113 × 10^7^.

### 3.4. KTD-YOLOv8 Model

**Figure 8:**
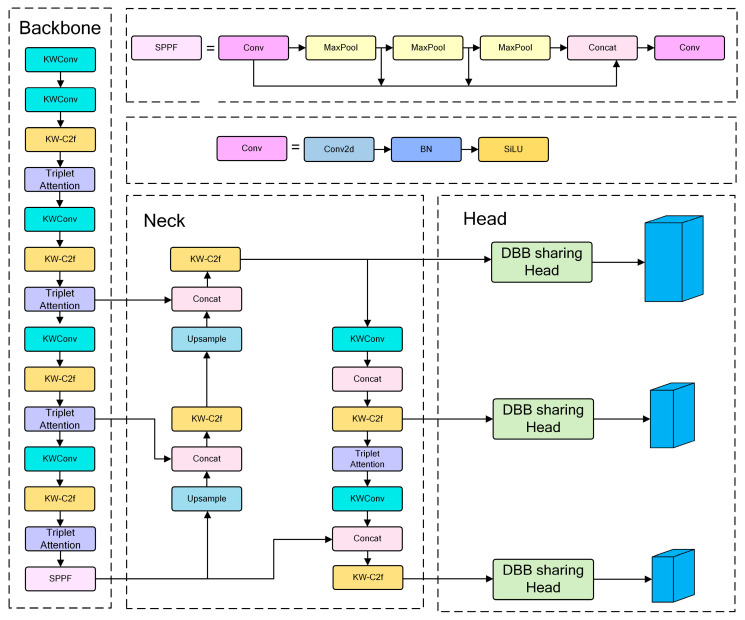
Diagram of KTD-YOLOv8 network structure.

Currently, YOLO networks are widely used in agriculture detection and segmentation tasks. YOLOv8 is characterized by fast detection and lightweight capability; thus, it is suitable for embedded devices. Compared with YOLOv5, YOLOv8 has advantages in several aspects. In the backbone network (Backbone) part, YOLOv8 adopts the DarkNet53 structure for a bottom–up feature extraction. It uses the C2f module to replace the C3 module of YOLOv5 and improves feature extraction efficiency and accuracy. The Path Aggregation Network (PANet) enhances the model’s feature fusion and multi-scale detection ability in the neck network part. In the head network part, YOLOv8 adopts a decoupled head structure to process the classification and detection tasks separately. This reduces complexity and improves accuracy. However, the actual strawberry leaf disease detection task is often affected by background interference, which leads to a larger computational load. Because of the limited calculation resources on embedded devices, it is difficult to achieve a balance between accuracy and computational load when using the original YOLOv8. In order to solve this problem, this study introduces three improvements based on the YOLOv8s model. Firstly, in the backbone network, KWConv is taken to replace the Conv function in YOLOv8, and KW-C2f is adopted to replace the C2f function in YOLOv8. Secondly, after each KW-C2f operation, the Triplet Attention mechanism is added. Thirdly, a DBB sharing head is constructed for parameter sharing. These improvements aim to improve the recognition accuracy and keep the model lightweight. The network structure of KTD-YOLOv8 is shown in Figure 8.

Each improving component is described below:

1. KernelWarehouse convolution (KWConv)

**Figure 9:**
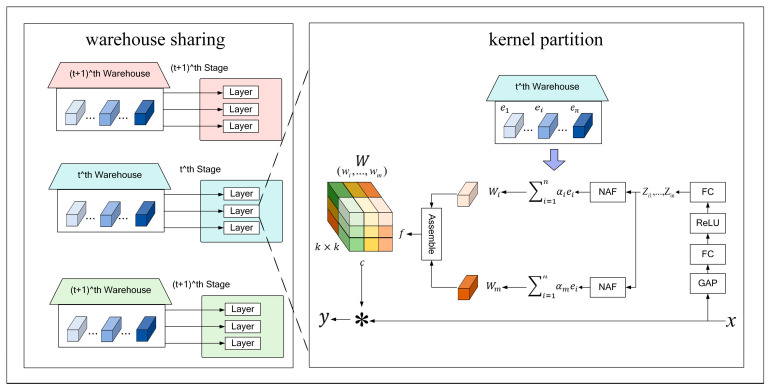
The framework of the KernelWarehouse dynamic convolution.

The structure of the KernelWarehouse convolution is shown in Figure 9. Its operation can be divided into two steps: kernel partition and warehouse sharing. In the kernel partition step, KernelWarehouse first divides the static kernel *W* in the convolution layer into *m* non-overlapping parts equally (*m* is set to 16 in this paper), *w*_1_, …, *w_m_*, which are called “kernel units”. Through these kernel units, kernel partition can be defined as

(3) $$W=w_{1}\cup \ldots \cup w_{m},i,j\in \left\{1,\ldots ,m\right\},i\neq j,w_{i}\cap w_{j}=\emptyset$$

In order to generate a high degree of freedom, a kernel partition is used to replace the kernel unit in the static convolution kernel *W*. Then, they are combined to form a new convolution kernel called KWConv. The linear mixture of kernel units is generated as follows. The core units *w*_1_, …, *w_m_* are regarded as the “local core”, and the “warehouse” E = {*e*_1_, …, *e_n_*} containing n core units is defined, where the dimensions *e*_1_, …, *e_n_* are the same as *w*_1_, …, *w_m_* and each core unit can be defined as

(4) $$w_{i}=\alpha_{i1}e_{1}+\ldots +\alpha_{in}e_{n},i\in \left\{1,\ldots ,m\right\}$$

where *α_i_*_1_, …, *α_in_* are the scalar attention elements generated by the new attention module (NAF) based on the input *x*.

For the *i*-th kernel unit in the static convolution kernel *W*, the attention function can be defined as

(5) $$\alpha_{ij}=\left(1-\tau \right)\frac{Z_{ij}}{\sum_{p=1}^{n}\left|Z_{ip}\right|}+\tau \beta_{ij},j\in \left\{1,\ldots ,n\right\}$$

where *τ* is a temperature parameter that decreases linearly from 1 to 0 during the training phase; *β_ij_* is a binary value (0 or 1) used to initialize attention. The feature scalars *Z_i_*_1_, …, *Z_in_* is generated as follows. Firstly, for any convolutional layer with a static convolution kernel *W*, a global average pooling (GAP) operation is performed to map the input to a feature vector. Then, it passes through a fully connected (FC) layer, a rectified linear unit (ReLU), another FC layer, and a new attention function. The length of the feature vector is reduced to 1/16 of the original one through the first FC layer. The *m* groups of feature scalars, named *Z_i_*_1_, …, *Z_in_*, are generated through the second FC layer. 

**Figure 10:**
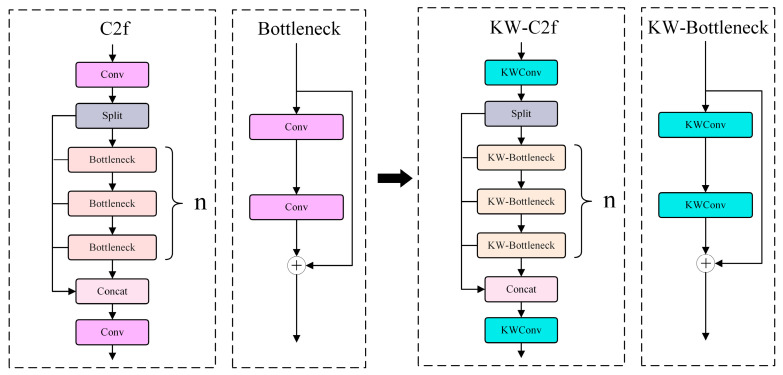
The structure of C2f, Bottleneck, KW-C2f, and KW-Bottleneck.

In the warehouse sharing operation, if the same convolution kernel unit size is used, multiple adjacent convolutional layers of KWConv can share the same warehouse value. This can enhance its efficiency and representation capability. In the YOLOv8 network, C2f function can reduce the resolution of the feature map and extract more representative features. In the components of the C2f module, the Bottleneck part can reduce the computation by decreasing channel numbers in the feature map, as shown in Figure 10. The base convolution in the C2f and Bottleneck parts are replaced by KWConv. The replaced C2f functions are named KW-C2f and KW-Bottleneck, respectively. KWConv reduces the computation by dynamically adjusting the weights of the convolution kernel. In addition, the KWConv can use the global average pooling and attention mechanisms to make the features extracted more representative.

2. Triplet Attention mechanism

The attention mechanism can help the model filter the target region with important information from many irrelevant background areas. In the actual environment, the spots of strawberry leaves only occupy a small portion of the image, so a specific attention mechanism is needed to deal with the target object information. The Triplet Attention mechanism is selected in this work to process the feature map (*C × H × W*), where *C* is the number of channels, *H* is the height, and *W* is the width. 

The high branch of the mechanism is responsible for computing the attention weights of the channel dimension *C* and the spatial dimension *W*. It rotates the input features by 90 degrees counterclockwise, and the rotated features follow *F*∈*R^C×H×W^*. Then, a *Z*-pooling operation is performed. The *Z*-pooling operation maximizes and averages the input tensor along the *H*-axis, combines the features, and maintains the original shapes by using convolutional layers and Sigmoid activation functions. Finally, the output is rotated 90 degrees clockwise around the H-axis. The central branch is responsible for capturing the dependencies between the channel dimension C and the spatial dimensions *H* and *W*. This branch first interacts on the *W*-axis with the input feature *F* rotated 90 degrees counterclockwise, and the rotated features follow *F*∈*R^C×H×W^*. Then, *Z*-pooling and convolution operations are performed, and the attention weights are generated by a Sigmoid function. Finally, the output is rotated 90 degrees clockwise around the *W*-axis to maintain the original shape. The low branch is used to capture the dependencies between spatial dimensions. This branch maintains the input’s identity and performs *Z*-pooling and convolution operations. It can later generate the attention weights through the Sigmoid function. The *Z*-pooling formula is shown in Equation (6).

(6) $$Z-POOL\left(X\right)=[MaxPool_{0d}\left(x\right),AvgPool_{0d}(x)]$$

**Figure 11:**
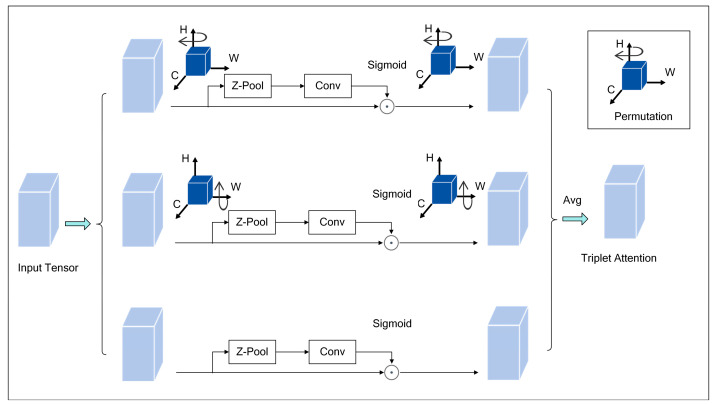
A schematic diagram of the Triplet Attention mechanism.

Each branch aligns the inputs after generating the attention weights (Permutation). Then, the outputs of the three branches are average aggregated (Avg) to obtain the Triplet Attention output. In this way, Triplet Attention can enhance the network’s attention to leaf spot diseases of strawberries and improve the efficiency and efficacy of the model. The schematic diagram of the Triplet Attention mechanism is shown in Figure 11.

3. DBB Sharing Head

**Figure 12:**
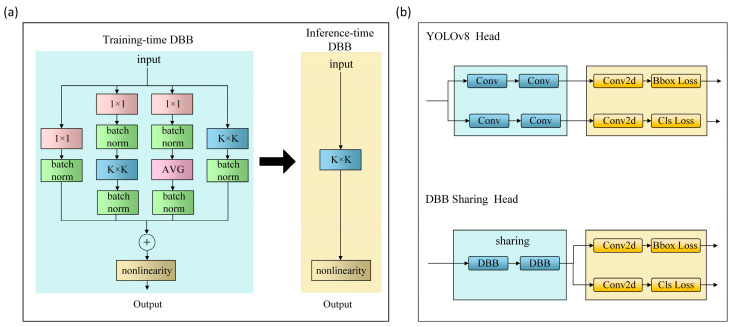
(**a**) The structure of the diverse branch block; (**b**) the structure of the YOLOv8 head with a DBB sharing head component.

Constructing a DBB sharing head aims to improve the feature extraction ability of the model and reduce the computational amount. DBB separates training from inference and uses a single convolution to combine different branches. During the training, DBB takes a multi-branch structure where each branch extracts different types of features. DBB uses 1 × 1, 1 × 1−*K* × *K*, 1 × 1-mean, and *K* × *K* instead of the regular *K* × *K* convolutions for feature extraction to augment the original layers. After extracting features from multiple branches, these features are fused to a final comprehensive value. Before the inference stage, reparameterization is performed in the multi-branch structure and fused into one main branch to save time. Because the DBB structure can be equivalently converted into a single convolutional layer for deployment, it can increase the training time to improve accuracy but has a relatively less impact on the inference time. The structure of the DBB training and inference process is shown in Figure 12a.

The YOLOv8 head structure and the DBB sharing head structure are shown in Figure 12b. DBB fuses multiple branches into one master branch under a multi-branch structure to extract different types of features before inference. Using the DBB to replace the original convolution of YOLOv8 can improve model accuracy without significantly increasing the computational complexity. Moreover, to further reduce the computational complexity, a parameter-sharing approach is constructed in this work. It uses two DBB modules to replace the four original convolutions in the decoupling head of YOLOv8 to improve efficiency.

## 4. Conclusions

In order to improve the recognition of strawberry leaf diseases, a KTD-YOLOv8 method is introduced in this work. Experimental results show that compared with the original YOLOv8, KTD-YOLOv8 improves the mAP@0.5 from 86.9% to 89.7%, reduces the model inference time from 13.1 ms to 12.1 ms, and reduces the calculation load from 28.8 GFLOPS to 17.7 GFLOPS, which is a reduction of 38.5%. Thus, it can achieve better performance in the recognition of strawberry leaf diseases with higher accuracy and lower computation, especially for mobile embedded devices. The detection results can help farmers detect and control the diseases early and gain more profits from growing strawberries.

## Figures and Tables

**Table 1:** Comparative results of ablation experiments.

Baseline	Convolution	Attention	Head	Accuracy/ %	Recalls/ %	mAP@0.5/%	GFLOPS	Parameters	Inference Time/ms
√				89.1	77.6	86.9	28.8	1.113 × 10^7^	13.1
√	√			91.3	77.2	87.9	14.2	1.123 × 10^7^	17.1
√		√		90.0	81.0	89.3	28.5	1.114 × 10^7^	14.2
√			√	89.2	79.4	88.1	31.8	1.327 × 10^7^	11.1
√	√		√	89.5	80.3	88.2	17.6	1.338 × 10^7^	12.3
√	√	√		92.1	79.0	89.2	14.3	1.124 × 10^7^	18.6
√		√	√	91.9	80.2	89.6	31.9	1.327 × 10^7^	11.8
√	√	√	√	90.0	81.3	89.7	17.7	1.343 × 10^7^	12.1

**Table 2:** Comparison of different base convolutions.

Convolution	Accuracy/%	Recall/%	mAP@0.5/%	GFLOPS
YOLOv8s	89.1	77.6	86.9	28.8
DySnakeConv	88.8	79.2	87.9	31.6
SPDConv	90.3	78.6	88.1	43.0
KWConv	91.3	77.2	87.9	14.2

**Table 3:** Comparative experiment for different detecting heads.

Head	Accuracy (%)	Recall (%)	mAP@0.5 (%)	GFLOPS
YOLOv8s	89.1	77.6	86.9	28.8
Aux Head	89.8	80.0	88.2	36.8
Pose Head	89.9	79.0	88.2	39.7
DBB Sharing Head	89.2	79.4	88.1	31.8

**Table 4:** Comparative experiment for different attention mechanisms.

Attention	Accuracy/%	Recall/%	mAP@0.5/%	GFLOPS
YOLOv8s	89.1	77.6	86.9	28.8
SimAM	88.1	79.9	88.7	28.4
CPCA	86.5	80.1	87.6	29.4
Triplet Attention	90.0	81.0	89.3	28.5

**Table 5:** Comparative results of different detection models.

Arithmetic	Accuracy (%)	Recall (%)	mAP@0.5 (%)	GFLOPS	Parameters	Inference Time (ms)
YOLOv5	86.9	77.8	86.5	14.2	0.711 × 10^7^	12.5
YOLOv6	86.5	73.6	83.2	44.0	1.629 × 10^7^	13.2
YOLOv7	89.8	78.7	88.0	103.2	3.650 × 10^7^	21.0
YOLOv8	89.1	77.6	86.9	28.8	1.113 × 10^7^	13.1
YOLOv9	89.2	80.0	89.4	237.7	5.097 × 10^7^	30.2
KTD-YOLOv8	90.0	81.3	89.7	17.7	1.343 × 10^7^	12.1

## Data Availability

The authors confirm that all data underlying the findings of this work are available within this manuscript. Raw data that support the outcome of this study are available from the corresponding authors, upon reasonable request.
